# A Viral Pilot for HCMV Navigation?

**DOI:** 10.3390/v7072801

**Published:** 2015-07-15

**Authors:** Barbara Adler

**Affiliations:** Max von Pettenkofer-Institute for Virology, Ludwig-Maximilians-University Munich, Pettenkoferstrasse 9a, 80336 Munich, Germany; E-Mail: adler_b@mvp.uni-muenchen.de; Tel.: +49-89-218072867; Fax: +49-89-218072902

**Keywords:** human cytomegalovirus, gH/gL glycoprotein complexes, virus navigation, UL148

## Abstract

gH/gL virion envelope glycoprotein complexes of herpesviruses serve as entry complexes and mediate viral cell tropism. By binding additional viral proteins, gH/gL forms multimeric complexes which bind to specific host cell receptors. Both Epstein–Barr virus (EBV) and human cytomegalovirus (HCMV) express alternative multimeric gH/gL complexes. Relative amounts of these alternative complexes in the viral envelope determine which host cells are preferentially infected. Host cells of EBV can modulate the gH/gL complex complement of progeny viruses by cell type-dependent degradation of one of the associating proteins. Host cells of HCMV modulate the tropism of their virus progenies by releasing or not releasing virus populations with a specific gH/gL complex complement out of a heterogeneous pool of virions. The group of Jeremy Kamil has recently shown that the HCMV ER-resident protein UL148 controls integration of one of the HCMV gH/gL complexes into virions and thus creates a pool of virions which can be routed by different host cells. This first mechanistic insight into regulation of the gH/gL complex complement of HCMV progenies presents UL148 as a pilot candidate for HCMV navigation in its infected host.

Epstein-Barr virus (EBV) and human cytomegalovirus (HCMV) spread in their hosts are considered to be highly directed. Current models how EBV or HCMV particles are navigated through the infected host are based on alternative gH/gL complexes in the virus envelope which target different host cells. EBV envelopes are complemented with dimeric gH/gL and trimeric gH/gL/gp42 complexes and HCMV envelopes with trimeric gH/gL/gO and pentameric gH/gL/UL128-131 complexes. Both EBV and HCMV show differences in cell tropism of virus progenies derived from different host cells. Several studies have addressed mechanisms of how host cells shape the tropism of virus progenies by influencing the gH/gL complement of virions.

For EBV, a switch in cell tropism of the virus derived from B cells or epithelial cells has been described [1,2]. Infection of B cells results in virus progeny rich in gH/gL which favors infection of epithelial cells, whereas infection of epithelial cells results in virus progeny rich in gH/gL/gp42 favoring infection of B cells. The likely mechanism for this switch in cell tropism is binding of gp42 to the EBV B cell receptor HLA class II in the endoplasmatic reticulum (ER) of B cells and targeting gp42 to degradation [1]. In contrast, epithelial cells, which do not express HLA class II, allow abundant formation of gH/gL/gp42 complexes. As a consequence, virus progeny from B cells is directed to epithelial cells and vice versa. *In vivo*, it has been shown that EBV particles shed in saliva are high in gp42 and may thus be directed to B cells, which are the first target cells in horizontal transmission of EBV [3].

For HCMV, differences in cell tropism of virus progenies released from different cell types cannot be explained so easily. Virions complemented with gH/gL/gO and gH/gL/UL128-131 can infect all HCMV host cells, including endothelial and epithelial cells, whereas virions complemented only with gH/gL/gO show a tropism restricted mainly to fibroblasts. Interestingly, virions lacking gH/gL/gO also show a massive loss of infectivity for epithelial and endothelial cells [4,5], which, as has been shown very recently, is due to gH/gL/gO also promoting fusion steps in gH/gL/pUL128-131-mediated entry [6]. However, this is only true for infection of cells with cell-free virus. Cell-associated spread in cell culture is not impaired when gH/gL/gO is lacking [4,5]. This is reminiscent of *in vivo* spread of gO-knockout mutants of murine cytomegalovirus (MCMV). Entry of gO-knockout mutants of MCMV into the first target cells is impaired, but subsequently shows a focal spread pattern in organs comparable to the wildtype (WT) virus [7]. Cell type-dependent differences in tropism of HCMV progenies have been described for virus progenies released from fibroblasts and endothelial cells [8]. Both fibroblasts and endothelial cells produce heterogeneous virus progenies consisting of virions complemented with gH/gL/gO and high or low amounts of gH/gL/UL128-131. Fibroblasts release the virions irrespective of their gH/gL/UL128-131 complement, whereas endothelial cells only release virus particles with few or no gH/gL/UL128-131 and no tropism for endothelial cells [8]. Cell type-dependent release of virus subpopulations may also account for the finding that HCMV released into urine and saliva of HCMV patients can infect fibroblasts but not endothelial cells [9]. In summary, virus progenies consisting of distinct populations with respect to gH/gL complexes would be crucial for a host cell-dependent routing of HCMV.

An elegant study by Li *et al.* [10] offers a mechanism of how the gH/gL complex complement of virions is created and an explanation how the complement of virions may contribute to directed virus spread. The authors identified the ER-resident HCMV UL148 protein as a regulator of incorporation of gH/gL/gO complexes into virions. If UL148 is deleted from the viral genome, incorporation of the trimeric gH/gL/gO complex into virions will be strongly impaired, resulting in a reduced capacity of virus particles to establish infection in fibroblast cultures and, when compared to infection of fibroblasts, an increased capacity to establish infection in epithelial cell cultures. This is reminiscent of the increased relative infection efficiency for endothelial cells which has been described for gO-knockout mutants of HCMV [8]. Data from Li *et al.* [10] also showed that UL148 only binds to gH/gL when gO or UL128 are not bound, which suggests that binding of UL148 may interfere with binding of gO or UL128 to gL [11]. Interestingly, UL130 and UL131 were also found in gH/gL/UL148 coprecipitates, a finding which made Li *et al.* [10] propose a model of reversible gH/gL/UL130/148 and gH/gL/UL131/148 complexes competing with formation of gH/gL/UL128-131 and directing gH/gL to formation of gH/gL/gO. This model lacks an explanation of why the absolute amounts of gH and gL in virions are drastically increased when UL148 is expressed. If gH/gL complexes bound gO instead of UL128-131, a shift in the relative amounts of gO and UL128-131 should be observed. Yet, considering the small amount of UL128-131 in virions [12], an increase in gO would be hardly detectable. Additionally, the amounts of gH and gL should not change at all. The presence of UL130 and UL131 in gH/gL/UL148 coprecipitates may just reflect binding of UL148 to intermediate gH/gL/UL130 and gH/gL/UL131 complexes formed during assembly of multimeric gH/gL complexes but not efficiently exported from the ER [13].

I would like to propose an alternative model which is also compatible with the data from Li *et al.* [10] (Figure 1). In this model, HCMV strain-specific properties of the constituents of the multimeric gH/gL complexes determine the relative numbers of gH/gL/gO and gH/gL/UL128-131 complexes being formed and integrated into virions in the absence of UL148 (Figure 1, pink area). Such strain-specific differences might, for example, account for the different capacities of UL148-knockout TB40 and AD169 to establish infection in fibroblast and epithelial cell cultures [10]. When UL148 is expressed, it reversibly binds to gH/gL and increases the affinity of gH/gL for gO, which leads to additional abundant formation of gH/gL/gO (Figure 1, blue area). The outcome is, as shown by Li *et al.*, a relative increase in infection in fibroblast cultures when compared to infection in epithelial cell cultures [10]. Due to abundant formation of gH/gL/gO, all virus particles produced in the presence of UL148 very likely comprise gH/gL/gO in their envelope, whereas incorporation of the less abundant gH/gL/UL128-131 complex into the viral envelope shows a Gaussian distribution. Thus, in the presence of UL148 highly infectious and - with respect to endothelial and epithelial cell tropism -heterogeneous virus progenies are created. Using a simplified depiction of virus progenies, Figure 2 offers a hypothesis, compatible with the data from Li *et al.* [10] of how UL148 might contribute to host cell-dependent routing of HCMV. Virions released from fibroblasts are 100% gO-positive when cells have been infected with WT virus, but only partially gO-positive when cells have been infected with ΔUL148 virus (Figure 2A). Consequently, WT virus infection is stronger in fibroblast cultures than in epithelial cell cultures, whereas infection with ΔUL148 virus is comparable for fibroblasts and epithelial cells [10]. Fibroblasts readily release virus particles complemented with gH/gL/UL128-131, whereas epithelial cells only release gH/gL/UL128-131-negative virions and direct gH/gL/UL128-131-positive virions to cell-associated spread [8] (Figure 2B). Thus, virus progenies released from fibroblasts and epithelial cells differ (Figure 2B, boxed area). Fibroblast-derived virus particles can infect all host cells, whereas epithelial cell-derived virus particles are restricted to target cells like fibroblasts. When UL148 is deleted, supernatant-driven and cell-associated spread in fibroblast cultures is reduced due to less virions complemented with gH/gL/gO. In epithelial cell cultures, gH/gL/UL128-131-dependent cell-associated spread is enhanced in the absence of UL148 whereas release of infectious virus particles targeting fibroblasts is strongly impaired.

**Figure 1:**
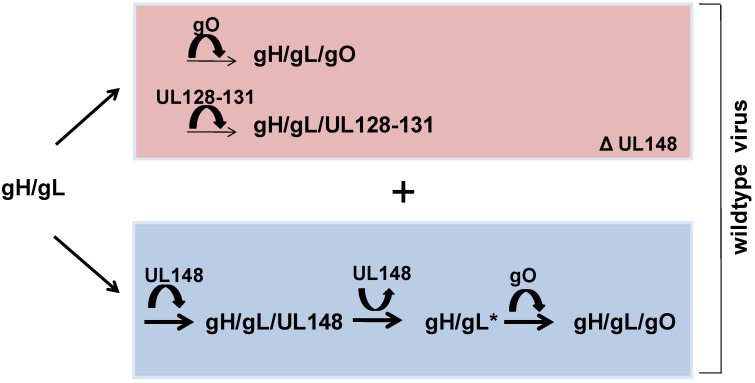
Model for a UL148-dependent regulation of alternative gH/gL complexes. HCMV strain-specific properties of the constituents of the gH/gL complexes determine the relative amounts of gH/gL/gO and gH/gL/UL128-131 complexes being formed and integrated into the viral envelope in the absence of UL148 (pink area). By reversibly binding to gH/gL, UL148 enhances binding of gH/gL to gO which results in an absolute increase in the amount of gH/gL/gO complexes in virions (blue area).

Do the findings from Li *et al.* [10] qualify UL148 as an important regulator of HCMV spread *in vivo*? A comparative sequence analysis of HCMV isolates has shown that UL148 is a highly conserved protein, which indicates that viral infection success *in vivo* is coupled to an intact UL148 [14]. Postulating a primary HCMV infection with cell-free virus particles, particles complemented with gH/gL/gO will, independently of the target cell types, be more successful in infecting first target cells than particles lacking gH/gL/gO [4,5,7]. By increasing the infectivity of virus particles released into body fluids, UL148 may contribute to the efficiency of horizontal transmission. Although fully speculative at the moment, cell type-dependent routing of HCMV in its infected host and to first targets of horizontal or vertical spread would depend on UL148-controlled production of heterogeneous and highly infectious virus progenies. Infection of Rhesus macaques with RH159 (orthologue of HCMV UL148) knockout mutants of RhCMV might be an appropriate animal model to elucidate the role of UL148 in virus spread *in vivo*.

**Figure 2:**
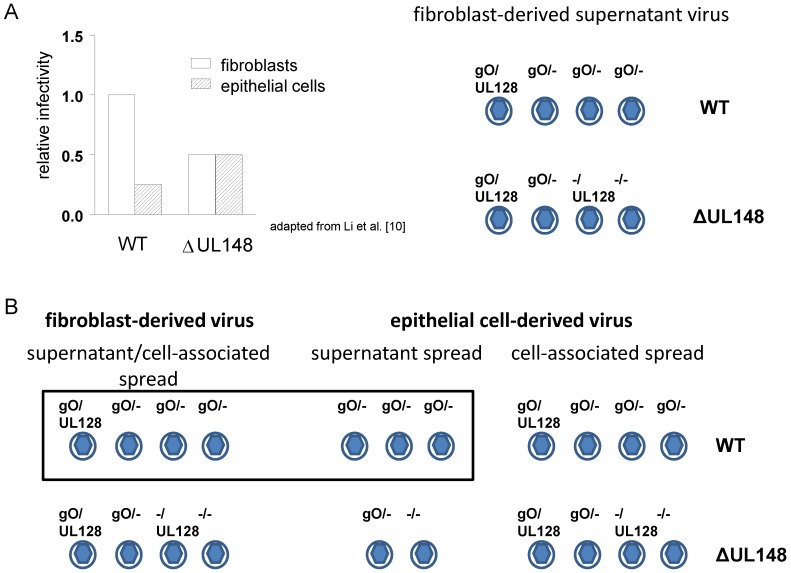
Model for a role of UL148 in shaping virus progenies. Based on differences in infection of fibroblasts and epithelial cells as described by Li *et al.* [10], a model of UL148-dependent shaping of virus progenies available for supernatant or cell-associated spread is proposed. Hypothetical and simplified virus progenies derived from fibroblasts infected with WT and ΔUL148 virus and the relative infectivities of these viral progenies are shown in (**A**); (**B**) shows a comparison of hypothetical virus progenies derived from fibroblasts and epithelial cells infected with WT or ΔUL148 virus. [gO/UL128] can infect fibroblasts and epithelial cells either cell-associated or via supernatant; [gO/−] can infect fibroblasts either cell-associated or via supernatant; [−/UL128] can infect fibroblasts and epithelial cells cell-associated, supernatant-driven infection of fibroblasts and epithelial cells is impaired; [−/−] not infectious.
